# Chromium Induces Toxicity at Different Phenotypic, Physiological, Biochemical, and Ultrastructural Levels in Sweet Potato (*Ipomoea batatas* L.) Plants

**DOI:** 10.3390/ijms232113496

**Published:** 2022-11-04

**Authors:** Sunjeet Kumar, Mengzhao Wang, Shah Fahad, Abdul Qayyum, Yanli Chen, Guopeng Zhu

**Affiliations:** 1 Key Laboratory for Quality Regulation of Tropical Horticultural Crops of Hainan Province, School of Horticulture, Hainan University, Haikou 570228, China; 2 Department of Agronomy, Abdul Wali Khan University Mardan, Mardan 23200, Pakistan; 3 Department of Agronomy, The University of Haripur, Haripur 22620, Pakistan

**Keywords:** sweet potato, chromium, photosynthetic pigments, stomatal traits, oxidative damage, antioxidant defense system

## Abstract

Crop productivity is enormously exposed to different environmental stresses, among which chromium (Cr) stress raises considerable concerns and causes a serious threat to plant growth. This study explored the toxic effect of Cr on sweet potato plants. Plants were hydroponically grown, and treatments of 0, 25, 50, 100, and 200 µM Cr were applied for seven days. This study exhibited that a low level of Cr treatment (25 µM) enhanced the growth, biomass, photosynthesis, osmolytes, antioxidants, and enzyme activities. However, significant deleterious effects in growth, biomass, photosynthetic attributes, antioxidants, and enzymes were observed at higher levels of Cr treatment. The remarkable reduction in plant growth traits was associated with the over-accumulation of H_2_O_2_ and MDA contents (410% and 577%, respectively) under the highest rate of Cr (200 µM). Under 200 µM Cr, the uptake in the roots were 27.4 mg kg^−1^ DW, while in shoots were 11 mg kg^−1^ DW with the highest translocation rate from root to shoot was 0.40. The results showed that the higher accumulation of Cr negatively correlated with the phenotypic and physiological parameters. It may be proposed that Cr toxicity causes oxidative damage as sustained by augmented lipid peroxidation, reactive oxygen species, and reduced photosynthetic rate, chlorophyll, and stomatal traits. The chloroplastic ultrastructure was damaged, and more apparent damage and size reduction were observed at higher Cr levels. Furthermore, aggregated Cr concentration positively correlates with the increase of osmolytes and superoxide dismutase (SOD) activity in the leaves of sweet potato. Moreover, improved osmolytes and SOD do not help protect sweet potato against high Cr stress. Overall, these findings will improve the understanding of the defense mechanisms of sweet potato to Cr stress.

## 1. Introduction

Heavy metals are a critical problem for plants, animals, and human health. Natural sources, industries, and excessive pesticides and fertilizers are the critical factors for heavy metal accumulation in the soil [1,2,3]. The contamination of heavy metals in agricultural land can lead to several health problems [4]. Among heavy metals, chromium (Cr) causes substantial soil and water contamination [5]. Chromium is the world 7th most abundant non-essential element in the world, and it is deposited in the soil and water by different natural sources and also through anthropogenic activities, including volcanoes, chromite, tanning, and other industrial emissions, such as electroplating, paints, mining, etc. [6,7,8].

Several countries, including China, Kazakhstan, India, and South Africa, are the world’s largest consumers of Cr [9]. In China, Cr and slag production has recently surpassed 400 million tons [10]. The emission of Cr in China from anthropogenic activities into the atmosphere has increased by 8.8% annually [11]. A previous study highlighted that Cr contents in the agricultural soil range from 1.48 to 820.24 mg/kg, much higher than the threshold (150 mg/kg) value [12]. This growing Cr release into water and soil may eventually cause a severe effect on plants, animals, and humans.

The Cr toxicity in plants depends on its mobilization, accumulation, uptake, and translocation [13]. Cr exists in different oxidation states, such as trivalent (Cr-III) and hexavalent (Cr-VI) states [14]. Cr-VI is reported to be more toxic than Cr-III because Cr-VI is more stable, highly water-soluble, and can easily penetrate the cell [3,8,15]. Cr stress triggers the production of reactive oxygen species (ROS), which lead to the impairment of cellular components and cause cell death [16,17]. The indications of Cr stress in plants include reduction in growth and biomass, photosynthesis rate, root cell damage, chlorosis, nutrient imbalance, alterations in enzymatic activities, and ultrastructural changes of chloroplast [9,16,18,19]. Additionally, Cr-treated plants accumulate Cr primarily in the roots and then translocate it to the shoots. Former studies showed that plants have their roots as the prime storing organ for heavy metals, whereas others exhibited toxicity tolerance in shoots [20,21]. Cr accumulation has reduced the germination rate, growth, biomass, photosynthetic pigments, and the enzymatic response of *Triticum aestivum* L., *Brassica napus*, and *Pisum sativum* L. [8,22,23].

Plants possess an antioxidant defense system to protect and recover from injuries due to oxidative stress and ROS under heavy metal stress. This defense system includes osmolytes (proline, soluble sugars, glycine betaine, and total proteins), antioxidants (carotenoids, glutathione (GSH), polyphenols, and flavonoids), and antioxidant enzymes (ascorbate peroxidase, peroxidase, catalase, and superoxide dismutase) [24,25,26]. A previous study on *Vigna unguiculata* plant reported an increment of proline content under Cr stress [27]. Another study reported that *Vigna radiata* and *Brassica juncea* seedlings exposed to high Cr stress enhance their antioxidant levels [5]. Similarly, the Cr toxicity on *Brassica napus* showed an increment in enzymatic activities [23,28]. These previous findings propose that an increment in the antioxidant defense system helps the plant to overcome heavy metal stress. Different studies reported that plants respond differently to Cr stress; for instance, *Nymphaea alba, Oryza sativa*, and *Cyamopsis tetragonoloba* are susceptible to Cr stress [29,30,31], whereas *Ocimum tenuiflorum, Jatropha curcas*, and *Bacopa monnieri* were found to be tolerant [18,32,33].

Sweet potato (*Ipomoea batatas* L.) is an important carbohydrate source in many regions of the world, especially in Asia and Africa [34]. All parts of the sweet potato are edible and used for different purposes; thus, the storage roots of sweet potato are targeted for ensuring food security, biofortification, and bioethanol production [35,36,37]. Similarly, the shoots and leaves of sweet potato are also used as green vegetables for human food and animal feed [38,39]. In addition, sweet potato leaves are rich in protein, minerals, fibers, vitamins, and phenolics and also possess medicinal properties [40,41,42]. Heavy metal stress markedly affects the growth, productivity, and quality of sweet potato [43]. Various studies have reported that a trace amount of metals can help stimulate the growth and production of horticultural crops [44,45]. Cr toxicity adversely affects metabolic processes in the plant, which ultimately reduces plant growth and production [46]. Different studies reported variations in the response of plants to Cr stress, and toxicity and tolerance level also varied in different plants. Cr toxicity and tolerance level vary within crop species, genotypes, and plant developmental stages. Conversely, limited research has been conducted on the effects of Cr toxicity in sweet potato, and the role of the sweet potato antioxidant defense system against the toxic effects of ROS under Cr stress has been poorly explained. Therefore, this study was designed to explore the effects of different Cr treatments on the phenotypic, physiological, and biochemical levels in the sweet potato plants and identify the Cr toxicity level of the sweet potato plant.

## 2. Results

### 2.1. Growth Parameters

**Figure 1:**
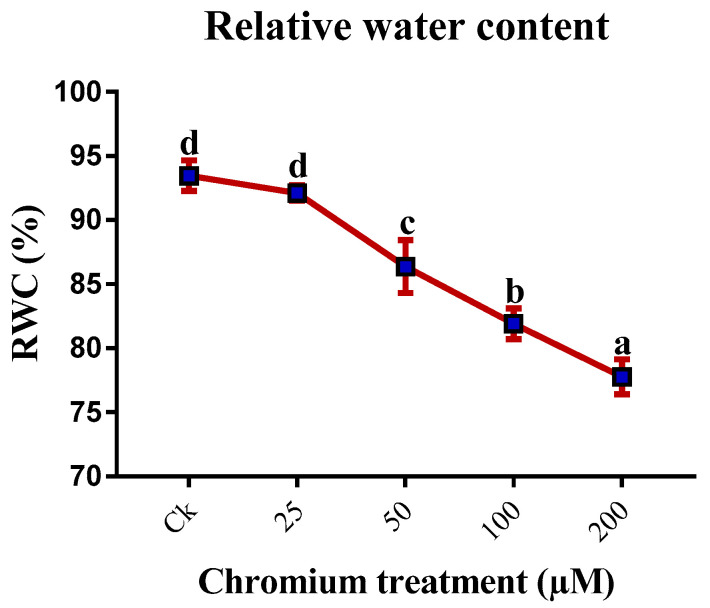
Effect of Cr on the RWC of the sweet potato. Means followed by different letters indicate a significant difference (*p* < 0.05) among the five treatments according to the Tuckey test. Error bars show mean ± SE.

The growth parameters of sweet potato significantly changed when applying different levels of Cr stress. The plant height, leaf area, number of leaves, shoot and root FW, shoot and root DW, root–shoot ratio, SDSI, and RDSI were found to be increased at 25 µM Cr treatment; conversely, a significant decrease (*p* < 0.05) was detected at 50, 100, and 200 µM Cr treatment as compared to the control (Table 1 and Table 2). At 25 µM Cr treatment, the plant height (15.8%), number of leaves (7.7%), leaf area (14%), shoot FW (7.9%), root FW (10.8%), shoot DW (5.7%), root DW (17.1%), SDSI (5.9%), and RDSI (17%) increased as compared to the control. Conversely, plant growth revealed a negative relationship at the higher level of Cr treatment. A maximum reduction in the plant height (49.3%), number of leaves (57.7%), leaf area (36.1%), shoot FW (48.3%), root FW (65%), shoot DW (59%), root DW (70.1%), SDSI (59.2%), and RDSI (70.2%) were detected at 200 µM Cr treatment (Table 1 and Table 2). Moreover, RWC was also found to be significantly decreased with the increase of Cr treatment, and the utmost decrease was recorded at 200 µM Cr (Figure 1).

### 2.2. Leaf Gas Exchange Elements

**Figure 2:**
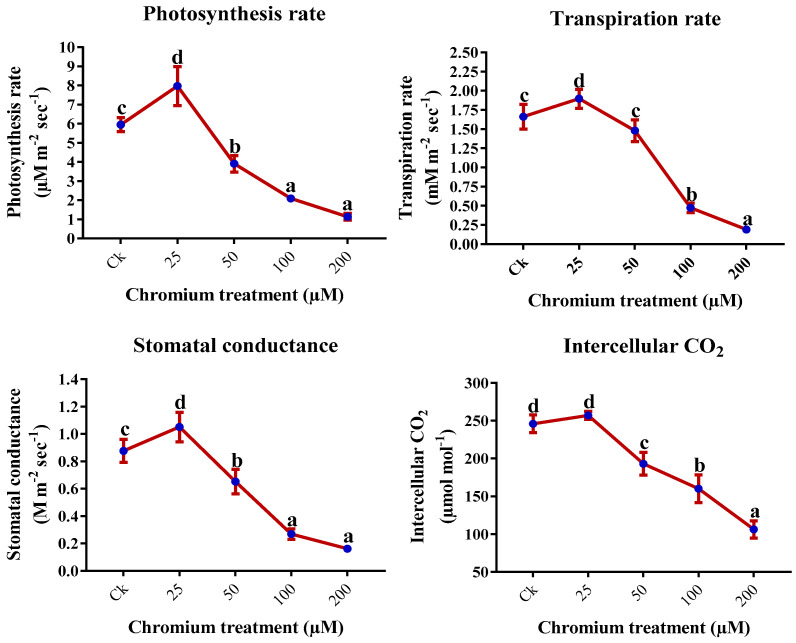
Impact of Cr on gaseous exchange elements in the leaf of sweet potato. Means followed by different letters indicate a significant difference (*p* < 0.05) among the five treatments according to the Tuckey test. Error bars show mean ± SE.

**Figure 3:**
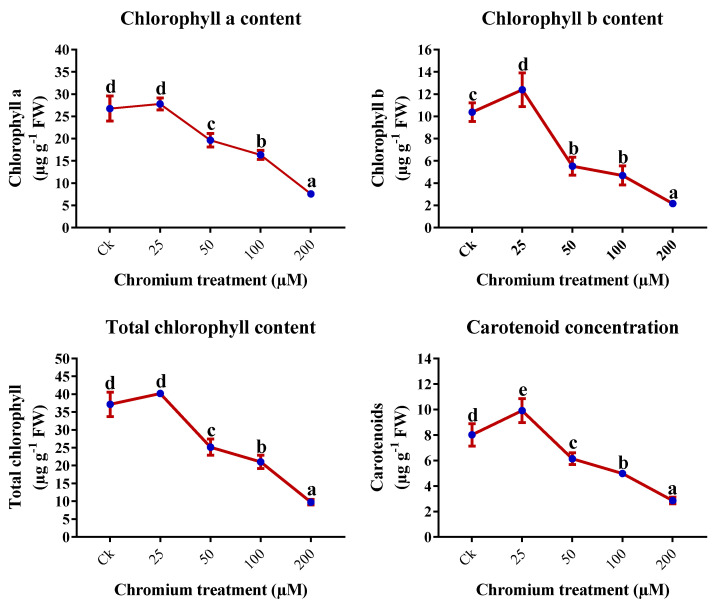
Impact of Cr on the photosynthetic pigments in the leaves of sweet potato. Means followed by different letters indicate a significant difference (*p* < 0.05) among the five treatments according to the Tuckey test. Error bars show mean ± SE.

The leaf gas exchange elements were improved significantly by the treatment of 25 µM Cr (*p* < 0.05). However, higher levels of Cr; 50, 100, and 200 µM exhibited a deleterious impact on the leaf gas exchange elements (Figure 2). Compared to the control, 25 µM Cr-treated plants showed 33.7%, 14.1%, 11.9%, and 4.5% increments in the photosynthesis rate, transpiration rate, stomatal conductance, and intercellular CO_2_, respectively (Figure 3). On the other hand, 80.9%, 88.5%, 81.5%, and 56.8% reductions were detected in the photosynthesis rate, transpiration rate, stomatal conductance, and intercellular CO_2_, respectively, when compared to the control (Figure 2).

### 2.3. Chlorophyll Content Analysis

The chlorophyll (Chl) content was significantly influenced by applying Cr stress. The content of the chl a, b, total chl, and carotenoids (Car) was increased minutely in 25 µM Cr-treated plants (Figure 3). On the other hand, the higher level of Cr treatment, 50, 100, and 200 µM, presented a deleterious impact on the photosynthetic pigments. Furthermore, 200 µM-treated plants exhibited an utmost reduction of 71.7%, 79.1%, 73.7%, and 64.3% in the chl a, b, total chl, and Car content, respectively, in comparison to the control (Figure 3).

### 2.4. Stomatal Structure Analysis

**Figure 4:**
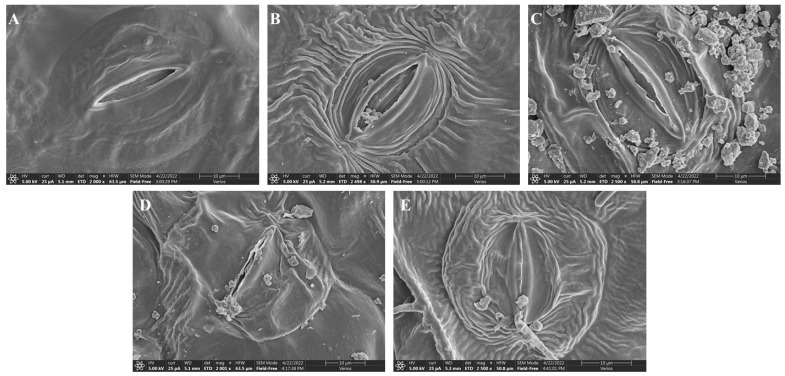
Impact of Cr on the stomatal traits in the leaves of sweet potato. (**A**) Ck, (**B**) 25 µM, (**C**) 50 µM, (**D**) 100 µM, and (**E**) 200 µM. Scale bar is 10 µm and magnification is (**A**) 2000×, (**B**) 2498×, (**C**) 2500×, (**D**) 2001×, and (**E**) 2500×.

The results showed that stomata size decreased with increased Cr treatment, and the maximum decrease was recorded at 200 µM Cr treatment (Figure 4). Compared to the control leaf, stomatal length under 200 µM Cr treatment was decreased by 53%; similarly, the width was reduced by 86.9%, pore length by 85.2%, and pore width by 94.7% (Table 3). The result revealed that Cr toxicity-induced stomata closing and reduced their size.

### 2.5. Root Morphology

**Figure 5:**
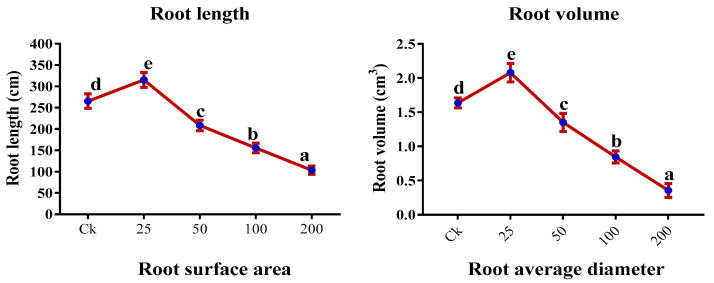

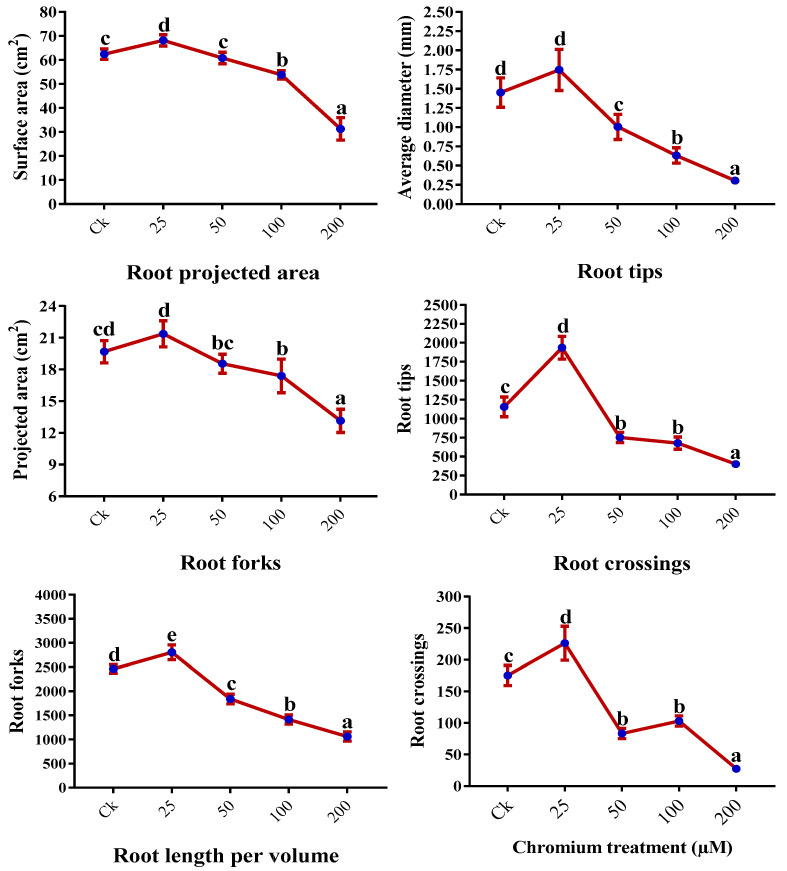

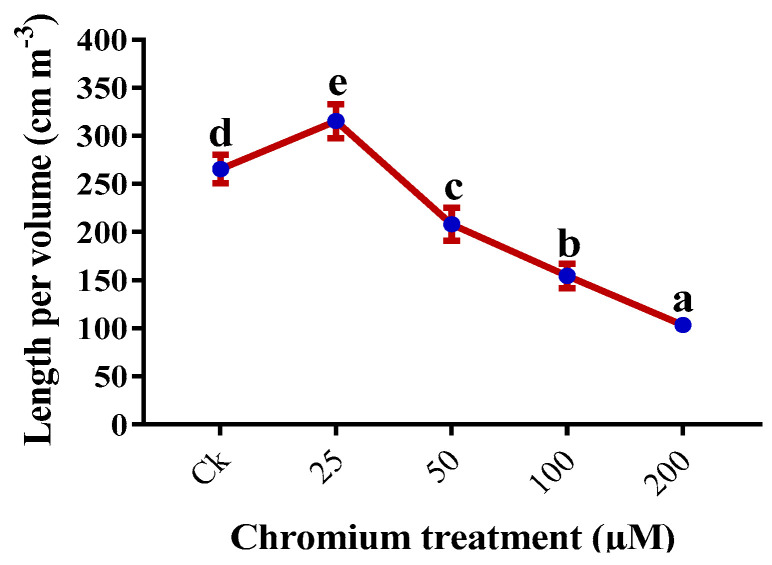
Impact of Cr on root morphological traits of sweet potato. Means followed by different letters indicate a significant difference (*p* < 0.05) among the five treatments according to the Tuckey test. Error bars show mean ± SE.

After 7 days of Cr treatment, the root growth of sweet potato was significantly enhanced at 25 µM Cr treatment (*p* < 0.05); conversely, 50, 100, and 200 µM Cr treatments presented a significant decrease in the root morphological traits (Figure 5). The increase in the root length was 18.7%; similarly, root volume showed 26.9%, surface area 9.2%, an average diameter of 20.4%, projected area of 8.6%, tips of 67.5%, forks of 13.8%, crossing of 29.1%, and length per volume of 18.7% increment in the plants treated with 25 µM Cr. In contrast, a significant reduction was observed from 50, 100, and 200 µM Cr treatments, and maximum reduction in root characteristics was detected at 200 µM Cr treatment. Root length (61%), root volume (78.3%), surface area (49.9%), average diameter (78.9%), projected area (33.2%), tips (65.3%), forks (56.9%), crossing (84.4%), and length per volume (61%) decreased under 200 µM Cr treatments when compared with the control (Figure 5).

### 2.6. H_2_O_2_ and MDA Content

**Figure 6:**
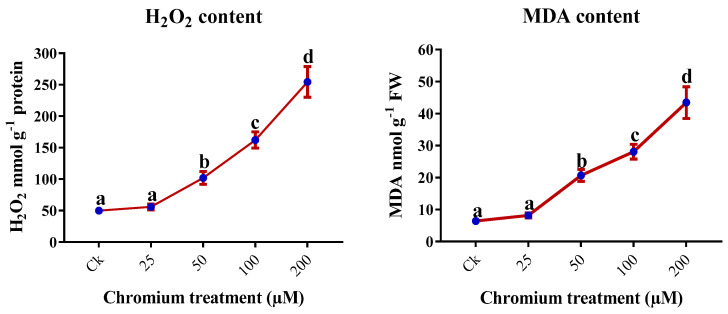
Impact of Cr on the production of hydrogen peroxide and MDA in the leaves of sweet potato. Means followed by different letters indicate a significant difference (*p* < 0.05) among the five treatments according to the Tuckey test. Error bars show mean ± SE.

The Cr treatment significantly triggered H_2_O_2_ and MDA contents in the leaves of sweet potato (*p* < 0.05; Figure 6). The increment in Cr stress increased the content of MDA and H_2_O_2_, and the maximum contents were detected in 200 µM Cr treatments as compared to the control. The H_2_O_2_ content in 200 µM Cr was 410.2% higher than the control, whereas the MDA content was 576.8% higher than the control (Figure 6).

### 2.7. Osmolytes and Antioxidants

Osmolytes, proline, and soluble sugars in the leaves of sweet potato were increased significantly with the increment of Cr treatment (*p* < 0.05). Compared to the control, the maximum increase of 342.2% in proline and 264% in soluble sugars were found in 200 µM-treated plants (Table 4). The total proteins were also found to be significantly increased with the increment of Cr treatment (*p* < 0.05) and maximum protein content was detected in 200 µM Cr treatment (Table 4). The content of GSH increased markedly at 25 µM Cr treatment (83.9% higher than the control), then it started to decrease, but the level of GSH at 50 and 100 µM Cr was still higher than the control (Table 4). The concentration of total polyphenols and flavonoids decreased significantly with increased Cr treatment (*p* < 0.05). Drastic effects in polyphenols and flavonoids were observed at 25, 50, and 100 µM Cr treatment, and the utmost reduction was recorded in 100 µM-treated plants. Surprisingly, an upsurge was detected in 200 µM Cr-treated plants but the concentrations were still much lower than the control (*p* < 0.05; Table 4).

### 2.8. Antioxidant Enzymes

**Figure 7:**
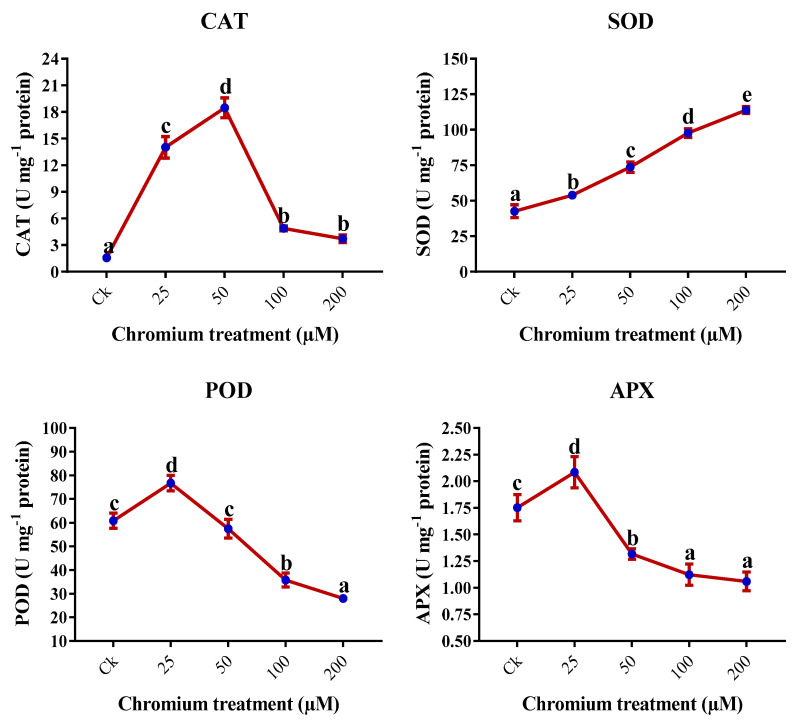
Impact of Cr on the antioxidant enzymes in the leaves of sweet potato. Means followed by different letters indicate a significant difference (*p* < 0.05) among the five treatments according to the Tuckey test. Error bars show mean ± SE.

Cr treatments have significantly influenced the antioxidant enzyme activities (*p* < 0.05). APX and POD activities increased significantly in 25 µM-treated plants and later decreasing at 50, 100, and 200 µM (*p* < 0.05), and maximum reduction was detected at the 200 µM Cr treatment. Compared to the control, a 39.5% decrease in APX and a 54% decrease in POD were observed at 200 µM Cr treatment (Figure 7). Similarly, the activity of the CAT was significantly increased to 50 µM and then started to decrease but the activity at 100 and 200 µM Cr was still significantly higher than in the control (*p* < 0.05; Figure 7). In contrast, the SOD was positively influenced by the Cr treatment, and the highest activity was detected at the 200 µM Cr treatment, which was 167.3% higher than the control (Figure 7).

### 2.9. Concentration, Uptake, and Translocation of Chromium

The results showed a positive correlation between Cr treatment and Cr concentration in both the shoots and roots of sweet potato. An increment in Cr application significantly increased the Cr concentration in both shoots and roots, the maximum concentration in the shoots was 105.46 mg kg^−1^ DW and in roots was 263.82 mg kg^−1^ DW (Table 5). Similarly, Cr accumulation was higher in the roots than in the shoots. Furthermore, Cr uptake by roots and shoots and translocation of Cr from roots to shoots increased significantly with the increased Cr level (Table 5).

### 2.10. Correlation and Principal Component Analysis (PCA)

**Figure 8:**
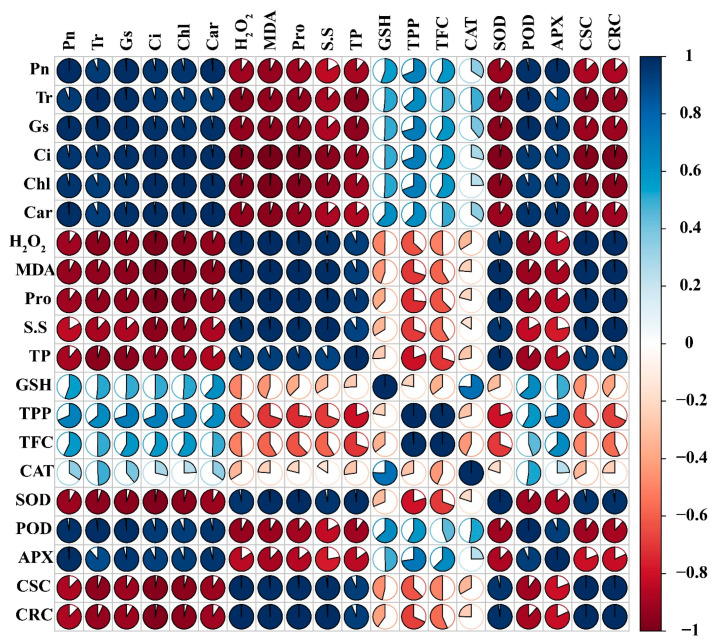
Pearson’s correlation analysis (*p* < 0.05) was measured between different physiological and biochemical traits of sweet potato. Pn (photosynthetic assimilation), Tr (transpiration rate), Gs (stomatal conductance), Ci (Intercellular CO_2_), Chl (total chlorophyll), Car (carotenoids), H_2_O_2_ (hydrogen peroxide), MDA (malonaldehyde), Pro (proline). S.S (soluble sugars), TP (total proteins), GSH (reduced glutathione), TPP (total polyphenols), TFC (total flavonoids), CAT, SOD, POD, APX, CSC (Cr shoot concentration), and CRC (Cr root concentration).

**Figure 9:**
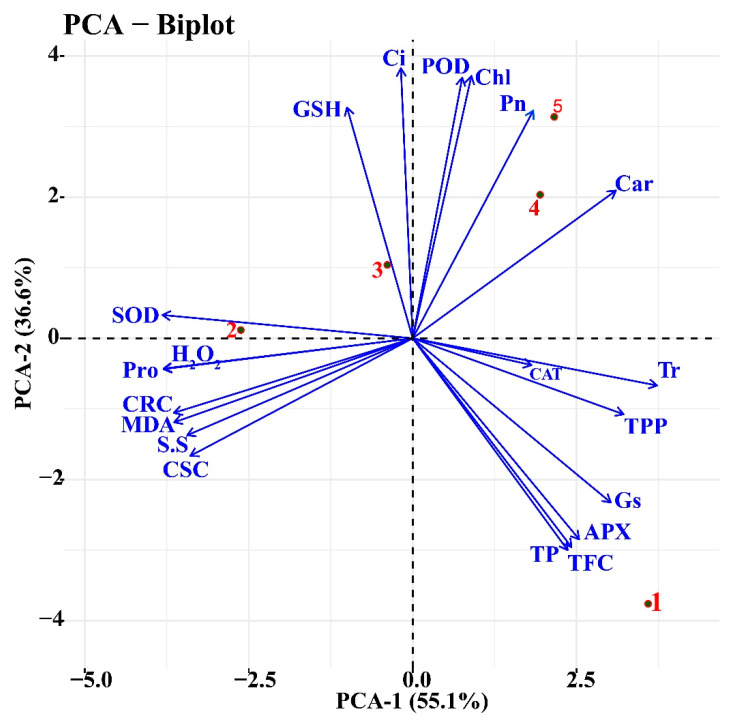
Principal component analysis of phenotypic, physiological, and biochemical traits under different levels of Cr treatments in sweet potato plant. Abbreviations are the same as mentioned in the legend in Figure 8. Red dots and numbers representing Cr treatments; (1) Ck, (2) 25 µM, (3) 50 µM, (4) 100 µM, and (5) 200 µM.

Pearson correlations and PCA showed significant correlations (positive and/or negative) in different Cr treatments and all plant physiological and biochemical parameters (Figure 8 and Figure 9). The variables that exist closely and in the same quadrant are positively correlated. Cr treatment at a higher level showed a significant decrease in physiological traits, and a negative correlation was found among physiological parameters and osmolytes, SOD, and Cr concentration (Figure 8). The chlorophyll content and photosynthesis assimilation were also negatively correlated with soluble sugars, proline, SOD, and concentrations of Cr (Figure 8). In contrast, a positive correlation was found among the growth parameters, chlorophyll content and photosynthesis assimilation, which illustrated that a decrease in photosynthetic rate is able to reduce the growth and development of the sweet potato plant (Figure 8). The PCA demonstrated obvious variations among morphological, physiological, and biochemical indices of the sweet potato (Figure 9). Blue vectors show a correlation among the studied parameters, while the red dots indicate different Cr treatments. A 91% variance was observed for both principal components, and PCA1 explained 55% and PCA2 36% for the mentioned plant growth attributes (Figure 9). The vectors of antioxidants and osmolytes, such as SOD, POD, soluble sugars, and proline, exhibited minute angles (<90º) with ROS, including MDA and H_2_O_2_, which suggest the strong relationship among the biochemical traits of sweet potato under chromium toxicity.

## 3. Discussion

Cr toxicity has rigorously affected agricultural land due to the continuous release of Cr wastes from industries into the environment, severely affecting the plants. Cr causes changes in various physiological and biochemical processes, resulting in increased or decreased metabolite production required for plant growth [7]. However, its positivity or negativity depends on the plant variety, chemical formula, quantity, and usage recurrence [47]. Moreover, some elements at low concentrations enhance the growth and production of the plants but, conversely, at higher concentrations depict deleterious effects [48,49]. Therefore, we investigated the phenotypic, physiological, and biochemical responses of the sweet potato under different levels of Cr stress and studied their mechanisms underlying Cr toxicity.

In this study, the growth parameters were significantly influenced, including plant height, leaf area, number of leaves, shoots and roots FW and DW, and root traits. At a low level of Cr treatment (25 μM), we found an increase in growth parameters and root morphological traits; however, higher Cr concentrations (50, 100, and 200 μM) exhibited a significant reduction in the length, growth, biomass, root morphological traits, and survival (Table 1 and Table 2; Figure 5). Plants with higher Cr levels produced relatively shorter and fewer lateral roots with coralloid structures. This decrease in root morphological traits might be due to the inhibition of mitotic cell division by prompting chromosomal abnormalities [18,50]. A study by Singh et al. reported that a low-level treatment of Cr enhanced growth, biomass, and root traits in chickpea plants; however, higher levels drastically impacted the growth [7]. Similarly, other researchers reported that Cr treatment has markedly reduced the growth and biomass of *Chrysopogon zizanioides*, *Plantago ovata*, and *Oryza sativa* [18,51,52]. In the current study, we also found an increment in SDSI and RDSI at 25 μM treatment in sweet potato; however, a negative correlation was detected with a further increment of the Cr (50, 100, and 200 μM) in the growth medium. Cr drastically affected plant growth and development by impeding their important metabolic processes [53]. These molecular shifts can reason for stunted growth and biomass.

RWC is an easy, convenient, and reliable parameter to calculate plant stress. We found a decrease in the RWC in the leaves of sweet potato under different Cr treatments, presenting that sweet potato plants were under stress. Different studies exhibited a decrease in RWC in the leaves of barley and maize under Cr stress [54,55]. Sensitive plant species cannot retain optimum water levels, which affects the osmotic adjustment of the plant. Kumar et al. also reported that stress conditions reduce RWC in plants [25].

Usually, the increment in chlorophyll content illustrates the photosynthesis assimilation and growth of the plant. Chlorophyll content decrease under different abiotic stress [25,26]. In this study, photosynthetic pigments and assimilation were invariably influenced by Cr stress. The present study revealed that gas exchange characteristics and chlorophyll content were significantly enhanced at 25 μM Cr treatment, and a drastic reduction was observed at higher Cr treatments (Figure 2 and Figure 3). A higher level of Cr has a negative impact on the transpiration rate, photosynthetic assimilation, and physiological processes, which have an important role in energy production, matter, and its translocation, subsequently causing a reduction in the growth, biomass, and development of the plant [56,57]. Decreased photosynthetic pigments and photosynthetic assimilation by Cr in *Oryza sativa*, *Brassica napus*, and *Zea mays* have also been reported [58,59,60]. The negative impact of Cr on photosynthetic pigments and gas exchange characteristics in several plants has been studied, endorsing that Cr affects the electron transport chain, membrane permeability, CO_2_ fixation, photosynthetic phosphorylation, chloroplastic ultrastructure, and electron diversions in the PSI [61,62]. This increase in ROS production also has an important role in reducing chlorophyll content [63].

Stomata are the vital factor that controls transpiration and CO_2_ transportation under different environmental stresses [64]. Previous studies mentioned that the decrease in the stomatal size is connected with more deformed stomata in the leaves under the heavy metals stresses, and stomatal closure is provoked by the interaction of the heavy metal with guard cells [65,66]. In this study, Cr toxicity initiated a decrease in stomatal length, width, pore length, and pore width (Figure 4 and Table 3). Purohit et al. reported that the application of Cr decreased the size of stomata in the leaves of eggplant and tomato [67]. The rise of heavy metals prompts cytotoxicity, hindering ionic absorption, lipid peroxidation, cell cycle arrest, and eventually causing cell death [9,68]. A decrease in stomatal size and closure can lead to deleterious effects on photosynthesis, transpiration, and gas exchange. This study revealed that alleviating the Cr stress caused a decrease in stomatal size and increased stomatal closure, which consequently reduces photosynthetic assimilation, transpiration rate, and gas exchange in the plant.

Different environmental stresses provoke lipid peroxidation. The increase in MDA content signifies the cell membrane injury and is considered a good indicator for evaluating abiotic stress [25]. ROS are generated by reacting heavy metals and fatty acids in plants [69]. Similarly, H_2_O_2_ is a component of ROS and increases with the increase in heavy metal stress. The present study illustrated that the Cr treatment significantly increased the MDA and H_2_O_2_ content in the leaves of sweet potato, and a maximum increment was observed in the 200 μM Cr treatment (Figure 6). Similarly, different studies also reported an increase in MDA and H_2_O_2_ content in the shoots of chickpea, vetiver, maize, and purslane [3,7,51,70]. The plant possesses an antioxidant defense mechanism to overcome this lipid peroxidation and ROS. In the current study, we found an increase in the contents of osmolytes (proline and soluble sugars). Generally, stressed plants produce higher osmolytes to protect their cells. Maize, chamomile, chickpea, and water dropwort plants displayed the same trend of increasing proline and soluble sugar contents in response to increased cellular dehydration under Cr stress [7,55,71]. Furthermore, proline and soluble sugars might not only protect the plant cellular membranes but also maintain turgor pressure, which eventually reduces the detrimental impact of Cr toxicity.

Total proteins act as osmotin and play a vital role in tolerance against abiotic stresses [25,72]. This study showed a significant increase in total proteins with increased Cr treatment (Table 4). These results agree with the *Chrysopogon zizanioides* and *Aeluropus littoralis* [51,73], which showed that total proteins were increased under Cr and other metals stresses. Rajendran et al. revealed that Cr treatment had synthesized new polypeptides, which might be linked with the genes induced by Cr stress [51]. These new polypeptides helped to improve the heavy metal tolerance in *Aeluropus littoralis*. The reduced glutathione (GSH) in plants can improve the tolerance against heavy metal stress. GSH is a good ROS scavenger in plants, which helps in the detoxification of free radicals [25]. The current study showed an increment in the GSH content under low Cr treatment; however, GHS started to decrease at a higher level, but the content of GSH was still higher than the control (Table 4). The results of this study agree with the findings of Kováčik et al. (2013) and Adhikari et al. (2020), which described the increment of GSH at the low level of Cr treatment and reduction at the higher level of Cr treatment [13,71]. Increased GSH under Cr application could be due to the increase in the activities of γ-glutamylcysteine synthetase and glutathione synthetase, which ameliorate tolerance against stress conditions [74]. Total polyphenols and flavonoids boost enzyme activities and act as antioxidants in stress conditions [26]. In the present study, we also found a significant reduction in polyphenols and flavonoid content under Cr stress (Table 4). Different studies reported a reduction in polyphenols and flavonoid content under heavy metals stress [75,76]. The decrease of polyphenols under Cr stress might be due to the interruption in the activity of different enzymes of phenylpropanoid pathways [76,77].

Antioxidant enzymes significantly reduce oxidative stress and ROS under environmental stresses [78]. This study showed variation in enzyme activities under different Cr treatments. This study showed an increase in the activity of SOD with the increase of Cr treatments. Similarly, CAT activity increased upto 50 μM then started to decrease; in contrast, POD and APX started to decrease after 25 μM Cr treatment (Figure 7). This increase in antioxidant enzymes could be due to the effect of the Cr ion on the production of free oxygen radicals. According to a previous study, chickpea treated with Cr showed throughout increment in the activity of SOD [7]. However, POD and CAT activities decrease after 90 μM and APX starts reducing after 60 μM Cr treatment. Similarly, Rajendran et al. highlighted the decrease in SOD and CAT increased under low concentrations of Cr (20 and 40 mg/L) in vetiver plants [51]. Rai et al. (2004) also exhibited an increment in the activity of CAT and a decrease in APX under Cr stress; however, SOD increased up to 20 μM Cr treatment and then decreased [32]. However, a decrease in the activities of different antioxidant enzymes was noted at the higher levels, which may be due to increased oxidative stress as a result of increased Cr toxicity. Similar results were observed in several plants exposed to Cr toxicity. The results of different studies on rice, Indian mustard, mungbean, and oilseed rape showed different responses of antioxidant enzymes under different Cr treatments [5,23,62]. The variation in antioxidant enzyme activity depends on the crop species, genotypes, and developmental stages of the plant and on the dosage, exposure time, and oxidation state of the heavy metals. Plant enzymatic activities play a vital role against ROS and regulate the cellular membranes under environmental stresses.

In this study, we found a positive correlation between Cr treatment and the concentration, uptake, and translocation of Cr in the roots and shoots (Table 5). Spinach, sunflower, pea, and maize exposed to Cr stress showed an elevated level of Cr concentration, and a higher concentration and uptake was observed in the roots [2,3,22,79]. The root is the primary organ of a plant that comes in contact with heavy metals, and this contact is the main reason for the higher concentration of Cr in the roots; thus, more Cr is absorbed and accumulated in the roots than in the shoots. Cr treatment in plants has increased ROS production, and ROS production was detected in plants treated with the highest level of Cr stress [3]. This study also found the same trend in ROS production and Cr stress. A previous study highlighted that Cr contents in the agricultural soil ranged from 1.48 to 820.24 mg/kg, much higher than the threshold (150 mg/kg) value. Similarly, Cr in surface water bodies ranges from 0.001 μg/L to 21.8 mg/L [12]. Furthermore, the normal range of Cr in plants ranges from 0.2 to 1 mg/kg DW [1]. This growing Cr release into water and soil may eventually cause a severe effect on plants, animals, and humans. In the present study, we found that shoots (edible part of the sweet potato) grown under normal condition have a Cr concentration of 0.26 mg/kg DW and uptake 0.03 mg/kg DW. However, it increased with the increase in Cr application. The highest dosage used (200 μM which is around 58.3 mg/L) in this experiment is considerably higher than the extreme concentration. Thus, our data demonstrated that starting at 15 mg/L and peaking at 58.3 mg/L, Cr causes severe damage to the growth of the plants.

## 4. Materials and Methods

### 4.1. Seedling Growth and Treatment

For this experiment, the “Haida HD7791” cultivar of sweet potato was used. Cuttings of the sweet potato were first disinfected with 1 g L^−1^ Carbendazim for 5–8 min and then grown in reverse osmosis (Ro) water till the appearance of roots. After that, the cuttings were transferred to half Hoagland nutrient solution (pH 5.8 ± 1) for a few days to adapt to the environment. To evaluate the Cr effect on sweet potato plants, hydroponic experiments were carried out in a controlled environment (25–27 °C for 16 h of photoperiod). The nutrient solution was replaced every 5 to 6 days to provide proper nutrients. Later, almost uniform size and healthy cuttings were subjected to each treatment. The seedlings of sweet potato were distributed into five equal groups in triplicates. Different concentrations of Cr (Potassium dichromate (K_2_Cr_2_O_7_)) were given to each group: 0, 25, 50, 100, and 200 µM. After 7 days of Cr stress, samples were collected for further analysis.

### 4.2. Growth Variables

The plant height, leaf area, number of leaves, and shoot and root fresh (FW) and dry weight (DW) were determined. The topmost leaves were used for the leaf area determination, and the data were collected using a portable laser leaf area meter (CI-202). The plant height of every plant was measured using a ruler. The FW of shoots and roots of the plants were calculated. After that, the calculated shoots and roots were placed at 70 °C for 3 days to determine the DW [25]. Shoot DW susceptibility index (SDSI) of the plant was calculated with the following formula:

SDSI = Shoot DW (_Stressed Plants_) × 100   Shoot DW (_Controlled Plants_) (1)

Similarly, the root DW susceptibility index (RDSI) of the plant was calculated with the following formula:

RDSI = Root DW (_Stressed Plants_) × 100   Root DW (_Controlled Plants_) (2)

### 4.3. Relative Water Content Analysis

To determine the relative water content (RWC), a protocol by Kumar et al. was used [25]. First, the leaves FW were recorded, then the leaves were immersed in ddH_2_O for four hours in a Petri dish; after that, the turgor weight of the immersed leaves were recorded. After that, the leaves were placed in the oven for one day at 70 °C to determine their dry weight. Finally, the RWC of the leaves was established with the following formula:

RWC% = [(FW − DW)/ (TW − DW)] × 100(3)

### 4.4. Gas Exchange Parameters and Root Morphology

To determine gas exchange parameters, completely developed leaves were analyzed using a portable photosynthesis system (CIRAS-3, Hansatech Co., USA) [80]. The roots of each plant were collected and washed with distilled water. Then, the roots were scanned with the help of the Imagery Scan Screen (Epson Expression 11000XL, Canada), and for the determination of root traits, WinRHIZO 2003a software was used [80].

### 4.5. Chlorophyll Measurement

Around 0.1 g of leaves were homogenized properly with 80% acetone. After that, the homogenized samples were centrifuged at 8000× *g* for 15 min and collected supernatant. The absorbance for chlorophyll a, b, and carotenoids were measured at 663, 646, and 470 nm using a full wavelength microplate reader (Infinite M200 PRO, TECAN, Swiss), respectively [81]. The concentration was determined with the following formula:

Chl *a* = 12.21(A663) − 2.81(A646) (4)

Chl *b* = 20.13(A646) − 5.03(A663) (5)

Car = [1000(A470) − 3.27(chl *a*) − 104(chl *b*)]/229 (6)

### 4.6. Scanning Electron Microscopy (SEM)

For this purpose, a previously published protocol with slight modification was followed [82]. The dried leaves were first acetylated for 2 to 3 min in 80% ethanol to eliminate any debris. The s-cutting was used to prepare tiny sections of leaf, then both abaxial and adaxial surfaces were fixed on the stub, sputtered with platinum for 25 min using Leica Mikrosystem GmbH, (ACE600), and finally studied with a scanning electron microscope (Thermo Scientific, Model: verios G4 UC) present in Hainan University, Haikou China.

### 4.7. Determination of MDA, H_2_O_2_, Proteins, GSH, and Antioxidant Enzymes

About 100 mg of fresh leaves were properly homogenized with 0.9 mL of 0.1 M phosphate buffer saline (PBS; pH 7.4). The homogenized samples were centrifuged at 5000× *g* for 15 min. The collected supernatant was utilized for the MDA quantification with the help of a kit (A003-1-1) purchased from Nanjing Jiancheng Bioengineering Institute, Nanjing, China. Finally, its absorbance was recorded at 530 nm [25].

About 500 mg leaves were properly homogenized with 4.5 mL of 0.1 M PBS, subsequently, the homogenized samples were centrifuged at 10,000× *g* for 15 min. The collected supernatant was utilized to determine the content of H_2_O_2_, total proteins, GSH, and antioxidant enzymes (CAT, POD, SOD, and APX) activities with the kits purchased from Nanjing Jiancheng Bioengineering Institute, Nanjing, China. H_2_O_2_ content was measured using H_2_O_2_ assay kit (A064-1-1), and its absorbance was recorded at 405 nm. Similarly, the A045-2 kit was used for total protein quantification, and its value of absorption was recorded at 595 nm. Likewise, the GSH content was quantified with a glutathione assay kit (A006-1), and its absorbance was recorded at 420 nm. Furthermore, the activities of enzymes were calculated using assay kits: CAT (A007-1), SOD (A001-1), POD (A084-3-1), and APX (A123-1-1). Their absorbance was recorded at 405, 550, 420, and 290 nm, respectively, and the activities were expressed as U mg^−1^ protein [25,26,83].

### 4.8. Determination of Proline and Soluble Sugars

For the determination of proline, an assay kit (A107-1-1) was used. About 0.1 g of fresh leaves were homogenized with the buffer provided by the company and followed the protocol provided by the company; finally, its value of absorption was determined at 520 nm. About 50 mg of fresh leaves were properly homogenized for the soluble sugars in 0.45 mL ddH_2_O. The homogenate-containing tubes were kept at 95 °C for 15 min then the tubes were cooled. Then, the homogenate was centrifuged for 15 min at 7500× *g* and the supernatant was collected. After that, the supernatant was diluted 10 times with ddH_2_O. The diluted extracts were used for the determination of soluble sugar content using a commercially available test kit (A145-1-1). Finally, its absorbance was recorded at 620 nm [25,26].

### 4.9. Determination of Total Polyphenols and Flavonoid Content

The protocol of Kumar et al. was used to quantify total polyphenols [81,84]. About 1 g of fresh leaf samples were crushed and homogenized with 60% ethanol. An amount of 1.25 mL of 10% Folin–Ciocalteu reagent was mixed with 0.25 mL of plant extract and 1 mL sodium carbonate decahydrate solution (0.75 g/mL). The mixture was incubated for about 15 min at 45 °C and then retained at room temperature for half an hour. Finally, its absorbance was recorded at 765 nm, and the data were presented against the gallic acid (GAE/g) standard.

For flavonoid content, about 0.25 mL of NaNO_2_ (0.5 g/mL) solution was mixed with 2 mL ddH_2_O and 500 µL of extract. The mixture was placed at 27 °C for 5 min. Then 150 µL of aluminum chloride solution (1 g/mL), 1 mL of sodium hydroxide (1 M) solution, and 1.2 mL of ddH_2_O were added concurrently. Finally, its absorbance was recorded at 510 nm, and the data were presented against the catechin (CAE/g) standard.

### 4.10. Cr Content Analysis

The concentration of Cr was measured using the wet digestion method. After seven days of treatment, the plants were cut, rinsed with Ro water, and dried to a constant weight. The 100 mg powdered plant samples were digested with 2 mL HNO_3_, 0.5 mL H_2_O_2_, and 1 mL deionized water using a super microwave-assessed digestion system (Anton Paar, Multiwave 7000, AUSTRIA). The content of Cr in the prepared samples was determined by ICP-MS (Perkin Elmer, NexION 5000G, USA) [25]. The uptake and translocation of Cr were calculated by the following formula:

Cr uptake (mg) = tissues Cr concentration × tissues dry mass(7)

Root to shoot Cr translocation = concentration of Cr in the shoots               concentration of Cr in the roots(8)

### 4.11. Statistical Analysis

All the phenotypic, physiological, and biochemical experiments were executed in triplicates. We used SPSS 25.0 software, and Tuckey tests were applied for the determination of significant differences (*p* ≤ 0.05) among the Cr-treated and control groups, and these differences were presented with different alphabets in the tables and figures. For the figures, GraphPad Prism 7 (San Diego, California, United States) was used. All the study results are represented as mean ± standard error (S.E). Principal component analysis (PCA) and Pearson correlations were performed using the “ggplot2” package in R (version 3.3.4, https://CRAN.R-project.org/package=ggplot2, accessed on 17 August 2022).

## 5. Conclusions

The widespread use of Cr in different industries causes environmental pollution. This Cr pollution has a negative impact on plants, including the sweet potato. Although the accumulation of osmolytes, antioxidants, and enzymes overcame oxidative stress and induced growth at a low concentration of Cr (25 uM), no signs and symptoms of oxidative stress were observed in the leaves. The current study identified that a low level of Cr benefits the growth and biomass of sweet potato. However, increased Cr accumulation did not maintain metal homeostasis. Cr stress increases MDA, H_2_O_2_, and Cr concentration and uptake in sweet potato and higher levels can cause Cr toxicity. The growth, biomass, photosynthetic assimilation, chlorophyll content, and stomatal traits of sweet potato were severely affected despite the rise in the osmolytes and SOD activities under high concentrations of Cr. The role of osmolytes, antioxidants, and enzymes may also be limiting. However, further studies are required at molecular levels to understand the tolerance mechanisms of Cr toxicity and their relationship with different metabolic processes.

## Figures and Tables

**Table 1:** Impact of Cr treatments on growth parameters of sweet potato.

Chromium (µM)	Height (cm)	Number of leaves	Leaf Area (cm^2^)	Shoot FW (g)	Root FW (g)
Ck	49.2 ± 3.4 ^c^	8.7 ± 0.6 ^c^	53.1 ± 3.2 ^c^	6.62 ± 0.4 ^d^	3.20 ± 0.5 ^cd^
25	57.0 ± 4.7 ^d^	9.3 ± 0.6 ^c^	60.5 ± 3.7 ^c^	7.14 ± 0.5 ^e^	3.54 ± 0.5 ^d^
50	44.5 ± 3.2 ^bc^	6.0 ± 1.0 ^b^	48.4 ± 5.0 ^b^	5.86 ± 0.7 ^c^	2.09 ± 0.2 ^bc^
100	39.6 ± 3.8 ^b^	4.7 ± 0.6 ^b^	42.5 ± 6.7 ^b^	4.31 ± 0.4 ^b^	1.60 ± 0.1 ^b^
200	24.9 ± 5.1 ^a^	3.7 ± 0.6 ^a^	33.9 ± 5.7 ^a^	3.43 ± 0.4 ^a^	1.12 ± 0.1 ^a^

Means followed by different letters indicate a significant difference (*p* < 0.05) among the five treatments according to the Tuckey test.

**Table 2:** Impact of Cr treatments on dry weight and susceptibility index of sweet potato.

Chromium (µM)	Shoot DW (g)	Root DW (g)	SDSI	RDSI
Ck	0.786 ± 0.09 ^d^	0.317 ± 0.02 ^d^	100	100
25	0.831 ± 0.08 ^e^	0.371 ± 0.04 ^e^	105.92 ± 2.6 ^d^	117.0 ± 2.8 ^d^
50	0.602 ± 0.06 ^c^	0.201 ± 0.02 ^c^	76.74 ± 2.5 ^c^	63.8 ± 9.5 ^c^
100	0.425 ± 0.05 ^b^	0.148 ± 0.02 ^b^	54.11 ± 0.6 ^b^	46.5 ± 4.4 ^b^
200	0.322 ± 0.05 ^a^	0.095 ± 0.01 ^a^	40.81 ± 2.4 ^a^	29.8 ± 0.9 ^a^

Means followed by different letters indicate a significant difference (*p* < 0.05) among the five treatments according to the Tuckey test.

**Table 3:** Impact of Cr on the stomatal traits of sweet potato leaf.

Chromium (µM)	Stomata Length (µm)	Stomata Width (µm)	Stomatal Pore Length (µm)	Stomatal Pore Width (µm)
Ck	26.19 ± 2.25 ^c^	16.15 ± 1.82 ^c^	19.47 ± 2.05 ^d^	3.94 ± 0.73 ^c^
25	25.68 ± 2.29 ^c^	14.97 ± 1.96 ^c^	19.15 ± 1.61 ^d^	3.65 ± 0.81 ^c^
50	18.43 ± 1.98 ^b^	8.88 ± 1.34 ^b^	11.54 ± 1.26 ^c^	1.51 ± 0.41 ^b^
100	15.35 ± 1.58 ^ab^	6.44 ± 1.07 ^b^	7.58 ± 1.24 ^b^	1.28 ± 0.23 ^b^
200	12.31 ± 1.13 ^a^	1.97 ± 0.55 ^a^	2.89 ± 0.62 ^a^	0.21 ± 0.08 ^a^

Means followed by different letters indicate a significant difference (*p* < 0.05) among the five treatments according to the Tuckey test.

**Table 4:** Impact of Cr on the production of osmolytes and antioxidants in the leaves of sweet potato.

Cr (µM)	Proline (µg g^−1^ FW)	S. Sugars (mg g^−1^ FW)	T. Proteins (mg g^−1^)	GSH (mg g^−1^ prot)	TPC (mg GAE g^−1^)	TFC (mg CAE g^−1^)
Ck	15.12 ± 0.99 ^a^	7.09 ± 0.46 ^a^	0.06 ± 0.00 ^a^	38.85 ± 1.89 ^a^	12.41 ± 0.92 ^d^	0.49 ± 0.04 ^c^
25	22.14 ± 1.74 ^b^	11.34 ± 1.01 ^b^	0.09 ± 0.01 ^ab^	71.44 ± 4.87 ^c^	9.61 ± 0.60 ^c^	0.35 ± 0.03 ^b^
50	35.49 ± 2.59 ^c^	14.29 ± 1.21 ^c^	0.11 ± 0.01 ^b^	58.11 ± 3.96 ^b^	7.17 ± 0.54 ^ab^	0.25 ± 0.01 ^a^
100	47.80 ± 4.02 ^d^	17.35 ± 1.25 ^d^	0.19 ± 0.02 ^c^	55.44 ± 3.06 ^b^	5.99 ± 0.43 ^a^	0.23 ± 0.02 ^a^
200	66.84 ± 4.79 ^e^	25.80 ± 1.46 ^e^	0.20 ± 0.02 ^c^	35.71 ± 2.40 ^a^	7.68 ± 0.74 ^b^	0.31 ± 0.02 ^b^

Means followed by different letters indicate a significant difference (*p* < 0.05) among the five treatments according to the Tuckey test.

**Table 5:** Impact of Cr in the content of Cr in the sweet potato.

Chromium (µM)	Concentration (mg kg^−1^ DW)		Uptake (mg kg^−1^ DW)		Translocation (Root to Shoot)
	Shoot	Root	Shoot	Root	
Ck	0.26 ± 0.02 ^a^	2.33 ± 0.23 ^a^	0.03 ± 0.00 ^a^	0.24 ± 0.02 ^a^	0.11 ± 0.02 ^a^
25	9.44 ± 0.62 ^a^	40.35 ± 2.06 ^b^	0.98 ± 0.05 ^a^	4.18 ± 0.15 ^b^	0.23 ± 0.00 ^b^
50	25.32 ± 1.16 ^b^	88.66 ± 9.03 ^c^	2.63 ± 0.14 ^b^	9.18 ± 0.80 ^c^	0.29 ± 0.03 ^c^
100	57.67 ± 5.20 ^c^	152.76 ± 13.94 ^d^	5.97 ± 0.45 ^c^	15.82 ± 1.22 ^d^	0.38 ± 0.01 ^d^
200	105.46 ± 12.73 ^d^	263.82 ± 7.63 ^e^	10.94 ± 1.41 ^d^	27.35 ± 1.11 ^e^	0.40 ± 0.04 ^d^

Means followed by different letters indicate a significant difference (*p* < 0.05) among the five treatments according to the Tuckey test.

## Data Availability

The raw data supporting the conclusions of this manuscript will be made available without any reservation.
